# Potency and Therapeutic THC and CBD Ratios: U.S. Cannabis Markets Overshoot

**DOI:** 10.3389/fphar.2022.921493

**Published:** 2022-06-06

**Authors:** Sarah D. Pennypacker, Katharine Cunnane, Mary Catherine Cash, E. Alfonso Romero-Sandoval

**Affiliations:** ^1^ Department of Anesthesiology, Pain Mechanisms Laboratory, Wake Forest University School of Medicine, Winston-Salem, NC, United States; ^2^ Department of Pharmacy, Wake Forest University School of Medicine, Winston-Salem, NC, United States

**Keywords:** cannabidiol, tetrahydrocannabinol, marijuana, medical marijuana, herbal cannabis, cannabis market, potency, intoxication

## Abstract

**Background and aims:** The effects exuded by cannabis are a result of the cannabinoids trans-Δ⁹-tetrahydrocannabinol (THC) and cannabidiol (CBD), and is dependent upon their pharmacological interaction and linked to the two cannabinoids’ concentrations and ratios. Based on current literature and trends of increasing cannabis potency, we postulate that most medical cannabis products with THC and CBD have ratios capable of producing significant acute intoxication and are similar to recreational products. We will test this by organizing products into clinically distinct categories according to TCH:CBD ratios, evaluating the data in terms of therapeutic potential, and comparing the data obtained from medical and recreational programs and from states with differing market policies.

**Methods:** We utilized data encompassing online herbal dispensary product offerings from nine U.S. states. The products were analyzed after being divided into four clinically significant THC:CBD ratio categories identified based on the literature: CBD can enhance THC effects (THC:CBD ratios ≥1:1), CBD has no significant effect on THC effects (ratios ∼ 1:2), CBD can either have no effect or can mitigate THC effects (ratios 1:>2 < 6), or CBD is protective against THC effects (ratios ≤1:6).

**Results:** A significant number of products (58.5%) did not contain any information on CBD content. Across all states sampled, the majority (72–100%) of both medical and recreational products with CBD (>0%) fall into the most intoxicating ratio category (≥1:1 THC:CBD), with CBD likely enhancing THC’s acute effects. The least intoxicating categories (1:>2 < 6 and ≤1:6 THC:CBD) provided the smallest number of products. Similarly, the majority of products without CBD (0%) contained highly potent amounts of THC (>15%). These results were consistent, regardless of differing market policies in place.

**Conclusions:** Despite the distinct goals of medical and recreational cannabis users, medical and recreational program product offerings are nearly identical. Patients seeking therapeutic benefits from herbal cannabis products are therefore at a substantial risk of unwanted side effects, regardless of whether they obtain products from medical or recreational programs. Efforts are needed to better inform patients of the risks associated with high potency cannabis and the interaction between THC and CBD, and to help shape policies that promote more therapeutic options.

## Introduction

Trans-Δ^9^-tetrahydrocannabinol (THC) and cannabidiol (CBD) are the two most prominent cannabinoids that comprise cannabis (Elsohly et al., 2014). The pharmacologic effects they each exude are quite distinct. For instance, CBD does not produce acute intoxication, has been proven to treat refractory epileptic syndromes in children, and may have anti-inflammatory, anxiolytic, and antipsychotic indications (Zuardi et al., 1993; Bergamaschi et al., 2011a; Bergamaschi et al., 2011b; Leweke et al., 2012; Iseger and Bossong, 2015; Devinsky et al., 2017). Yet, there is currently no substantial evidence that CBD alone has analgesic efficacy in humans—the primary indication for which patients seek out cannabis in the United States (U.S.) (Boehnke et al., 2019). On the other hand, THC produces the acute intoxication associated with cannabis and has been linked to multiple undesirable effects, such as paranoia, memory impairment, increased risk for psychotic illness, and cannabis dependency and the development of cannabis use disorder (CUD) (Di Forti et al., 2009; Izzo et al., 2009; Freeman et al., 2014).

Notably though, THC has shown promising analgesic efficacy (Abrams et al., 2007; Ellis et al., 2009; Ware et al., 2010; Wilsey et al., 2013; Wallace et al., 2015; Wilsey et al., 2016; van de Donk et al., 2019). This analgesic effect of THC is still under investigation (Boehnke and Clauw, 2019) but likely mirrors THC’s concentration and thus cannabis’ intoxication potential (Wilsey et al., 2013; Andreae et al., 2015; Wallace et al., 2015; van de Donk et al., 2019). In clinical trials studying the analgesic efficacy for cannabis, the THC concentrations utilized are consistently <10% (Abrams et al., 2007; Ellis et al., 2009; Ware et al., 2010; Wilsey et al., 2013; Wallace et al., 2015; Wilsey et al., 2016). In fact, significantly lower THC concentrations (1–3%) were used in several of the studies and resulted in sufficient clinical efficacy to manage pain (Wilsey et al., 2013; Wallace et al., 2015; Wilsey et al., 2016). Furthermore, adverse event potential and subsequent treatment discontinuation seems to increase at higher THC concentrations utilized in these studies. This parallel between THC concentration and intoxication and adverse event potential is increasingly becoming an issue as the potency of cannabis available rises (ElSohly et al., 2016; Chandra et al., 2019) despite patients often wishing to experience therapeutic benefits of THC without the associated subjective side effects (Joy et al., 1999; Grotenhermen, 2004; Hall, 2015). As a result, a difficult balancing act between analgesia and acute intoxication ensues.

Still, cannabis with high concentrations of THC (>15%) and greater intoxication potential is often favored in the recreational realm (Romero-Sandoval et al., 2018) and is associated with worse chronic pain in regular users (Boehnke et al., 2020). This discrepancy between the goals of medical and recreational products presumably should be reflected in the potency of the products each type of market offers. However, our previous findings demonstrated that average THC concentrations advertised online in medical programs are similar to those in recreational programs (Cash et al., 2020). Moreover, frequent medical cannabis users prefer inhaled cannabis with high levels of THC (Boehnke et al., 2020). The accessibility of high potency products could create a misconception about the safety of cannabis and downplay the risks and side effects associated with products containing high THC concentrations. It also leaves patients looking to use cannabis for medical purposes with mostly products outside the realm of what is considered potentially suitable for therapeutic purposes (Romero-Sandoval et al., 2018). It is important to note that while there may be some patients who enjoy the “high” or are willing to assume the risk of high THC consumption (as it may happen with opioids), this is not recommended from a medical standpoint. This sentiment is strongly supported by the International Association for the Study of Pain (IASP), which recently released a report which concluded that much more research is needed to determine the benefits and risks of cannabis for the treatment of pain before there is a chance cannabis can be endorsed for such usage (IASP Presidential Task Force on Cannabis and Cannabinoid Analgesia, 2021).

While these previous findings are certainly alarming, they only show a partial picture of the cannabis products offered in legal U.S. markets. CBD has long been proven to alter cannabis’ effects, and while CBD data was presented alongside THC concentrations categories in our previous study, the data was not thoroughly analyzed in relation to THC:CBD ratios and concentrations (Cash et al., 2020). Literature suggests that different concentrations of THC and CBD and different ratios of THC:CBD induce variances in experienced subjective effects (Pennypacker and Romero-Sandoval, 2020). In fact, it appears that certain lower ratios of THC:CBD are more apt to produce an attenuation of THC induced effects (Dalton et al., 1976; Englund et al., 2013; van de Donk et al., 2019) while higher ratios are more likely to enhance THC induced effects (Arkell et al., 2019; Solowij et al., 2019; van de Donk et al., 2019). For instance, one study found that inhaled cannabis at a 2:1 THC:CBD ratio (8 mg THC (1.6%)/4 mg CBD (0.8%)) enhanced the subjects’ intoxication when compared with THC alone (8 mg), but a 1:20 THC:CBD ratio (8 mg THC (1.6%)/400 mg CBD (80%)) reduced the subjects’ intoxication when compared with THC alone (8 mg) (van de Donk et al., 2019). Notably, these findings are counterintuitive to the popular idea that CBD is simply protective against the intoxicating effects of THC, that CBD is the yin to THC’s yang.

It is therefore important to determine whether the products available in dispensaries are pharmacologically safe for patients (medicinal) or the general public (adult use or recreational), not only by means of THC concentrations, but also CBD concentrations and the ratio of THC:CBD. Our previous findings clearly show that when analyzing the types of products offered in legal cannabis markets based solely on THC, the majority of products contain levels not recommended (i.e., >15% THC) since they are associated with strong intoxicating effects (Cash et al., 2020). However, we wonder whether the combination of these high THC levels with certain CBD concentrations and/or the ratio of THC:CBD could result in a pharmacological interaction that reduces the risk of high levels of THC. We identified some products that are more pharmacologically amenable to medical purposes, based on their THC levels (i.e., <10%) (Cash et al., 2020); but it is also possible that these products will contain THC:CBD ratios that lead to a pharmacologic interaction that enhances THC intoxicating effects (Pennypacker and Romero-Sandoval, 2020). In other words, it is clinically relevant to garner whether or not the existing products contain these two cannabinoids at concentrations and ratios that are suitable for patients. Specifically, it is necessary to determine if CBD at the levels available in dispensaries will exude pharmacologic protective/beneficial effects or detrimental effects to established THC liabilities (e.g., stronger intoxication, withdrawal, tolerance, dependence, addiction, psychiatric issues, etc.).

Since medical cannabis programs mimic recreational programs, we hypothesize that ratios and concentrations of CBD in products available in medical cannabis programs are similar to that in recreational cannabis programs, with the majority at levels which will likely enhance THC’s subjective effects. This study subsequently will test this hypothesis following these aims: 1) identify and categorize the THC:CBD ratios associated with different clinically meaningful pharmacologic effects when administered in conjunction via inhalation, 2) characterize the cannabis products available online within the determined ratio categories, 3) evaluate whether the probable pharmacologic effects of products labeled as recreational differ from the probable effects of products labeled as medical, and 4) determine if varying types of market structures (e.g., medical and recreational products offered in same facility or in separate facilities) provide clinically different cannabis offerings based on THC:CBD ratios.

## Materials and Methods

### Data Collection

We utilized the publicly available data set from our previously published study (Cash et al., 2020). To summarize, states with legalized medical and/or recreational programs that have legalized cannabis for pain management were identified. The data sampling included online dispensary product offerings from nine U.S. states and spanned two distinct geographical locations: the Northeast region [Maine (ME), Massachusetts (MA), New Hampshire (NH), Rhode Island (RI) and Vermont (VT)] and the Western region [California (CA), Colorado (CO), New Mexico (NM), Washington (WA)] of the United States (U.S.). At the time of sampling, all of the Northeastern states as well as NM had legalized only the medical use of cannabis, and CA, CO, and WA had legalized cannabis for both medical and recreational use. Additionally, medical and recreational products were offered in separate facilities in WA, while both medical and recreational products were allowed to be offered in the same building in CO, and products were not differentiated medical or recreational in CA. Inhaled cannabis has a more favorable pharmacokinetic profile than other routes of administration and has shown analgesic efficacy for various chronic pain conditions, the most common reason cited for seeking out medical marijuana in the U.S. (Wilsey et al., 2013; Andreae et al., 2015; Wallace et al., 2015; Romero-Sandoval et al., 2018). Herbal products (flowers and pre-rolls) were therefore the focus of the sampling. Individual product cannabinoid data (THC and CBD content) was recorded.

### Ratio Categorization

In order to carry out the first study aim, and based on our previous observations (Pennypacker and Romero-Sandoval, 2020), we identified four clinically significant THC:CBD ratio categories: CBD can enhance THC effects (THC:CBD ratios ≥1:1), CBD has no significant effect on THC effects (ratios ∼1:2), CBD can either have no effect or is protective against THC effects (ratios 1:>2 < 6), or CBD is protective against THC effects (ratios ≤1:6). Products with THC:CBD ratios >0.7 were considered to fall into the first category, ≥1:1. Products with THC:CBD ratios ≥0.4 and <0.7 were considered to fall into the second category, ∼ 1:2. Products with THC:CBD ratios <0.4 and >0.167 were considered to fall into the third category, 1:>2 < 6. And finally, products with THC:CBD ratios ≤0.167 were considered to fall into the fourth category, ≤1:6. While further investigation into concomitant administration of THC and CBD, their pharmacological interaction, and the resulting effects is certainly needed, this theme remained consistent throughout a thorough review of the literature (Pennypacker and Romero-Sandoval, 2020).

### Statistical Analysis

Mean and standard deviation analysis was performed for each state and for distinct medical and recreational program comparisons. The four clinically significant THC:CBD ratio categories, ≥1:1, ∼1:2, 1:>2 < 6, and ≤1:6, were analyzed for each state and program type. Either Student’s T test or One-way ANOVA and Turkey’s multiple comparison test were used, and a *p* < 0.05 was considered statistically significant. Relevant data is presented as (mean ± SD; median 25% percentile, 75% percentile).

## Results

**FIGURE 1:**
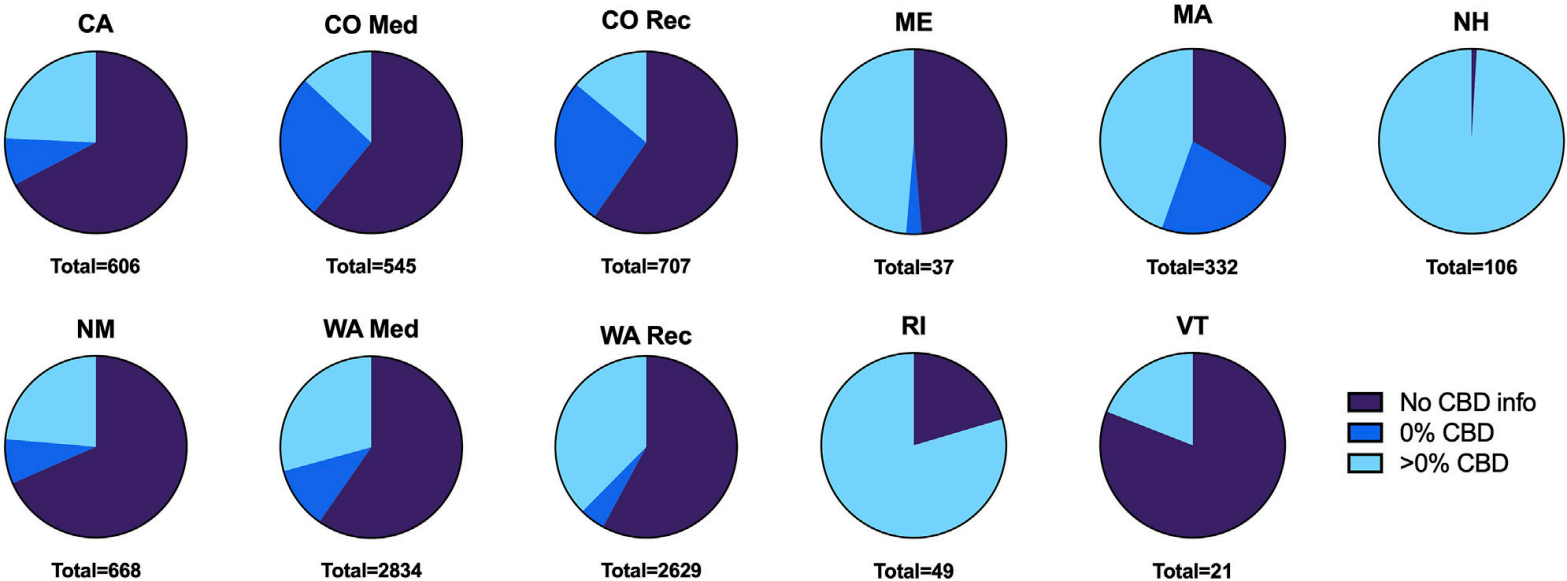
Proportion of products by CBD content information provided per state. Total products sampled per state listed below each graph.

Our results come from 8,534 herbal cannabis products (we did not exclude any product based on THC concentration) and their THC and CBD concentration information (Cash et al., 2020). These products were obtained from 653 dispensaries’ websites from nine states; CA (*n* = 606 total products), CO (*n* = 545 for medical, *n* = 707 for recreational), ME (*n* = 37), MA (*n* = 332), NH (*n* = 106), NM (*n* = 668), RI (*n* = 49), VT (*n* = 21), WA (*n* = 2,834 for medical, *n* = 2,629 for recreational). We found that most of these products, 58.5%, do not have any CBD content information. Of the 3,545 products with CBD content information (41.5% of all surveyed products), 839 (26.5% of products with CBD information) reported 0% content and 2,606 (73.5% of products with CBD information) reported >0% CBD concentration . The proportion of products with no CBD content, with 0% CBD content, and with >0% CBD content information varies widely among states (Figure 1).

**FIGURE 2:**
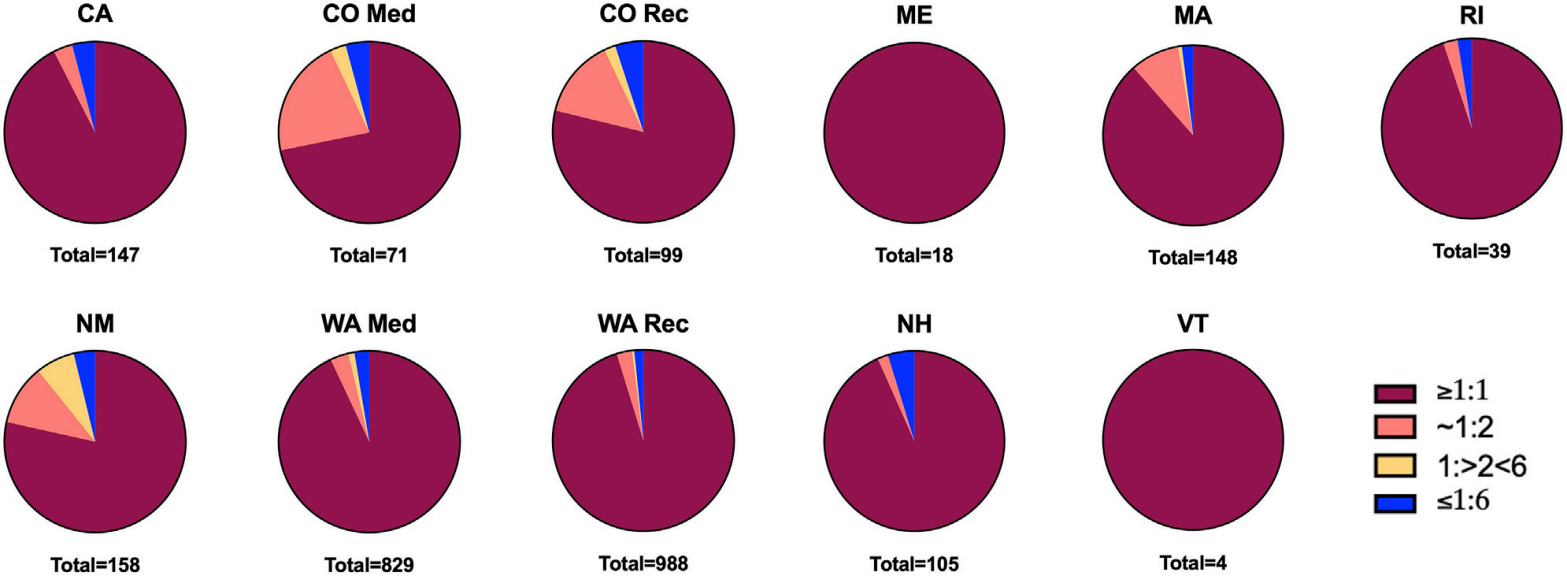
Proportion of products within THC:CBD ratio categories per state. Total products per state listed below each graph.

For subsequent analysis, we used only products with >0% CBD content, unless otherwise indicated. We noticed that not all evaluated states offered products belonging to all four THC:CBD categories we consider clinically meaningful. However, all the states offer products from the THC:CBD ratios ≥1:1 category (CBD enhances THC effects, Figure 2). In fact, and in line with our hypothesis, the majority of both medical and recreational products analyzed (72–100%) fall into the foremost listed category, with CBD likely potentiating THC effects; and products likely to provide CBD mitigation of THC effects (THC:CBD ratios ≤1:6) make up the smallest category (0–5%, Figure 2).

**FIGURE 3:**
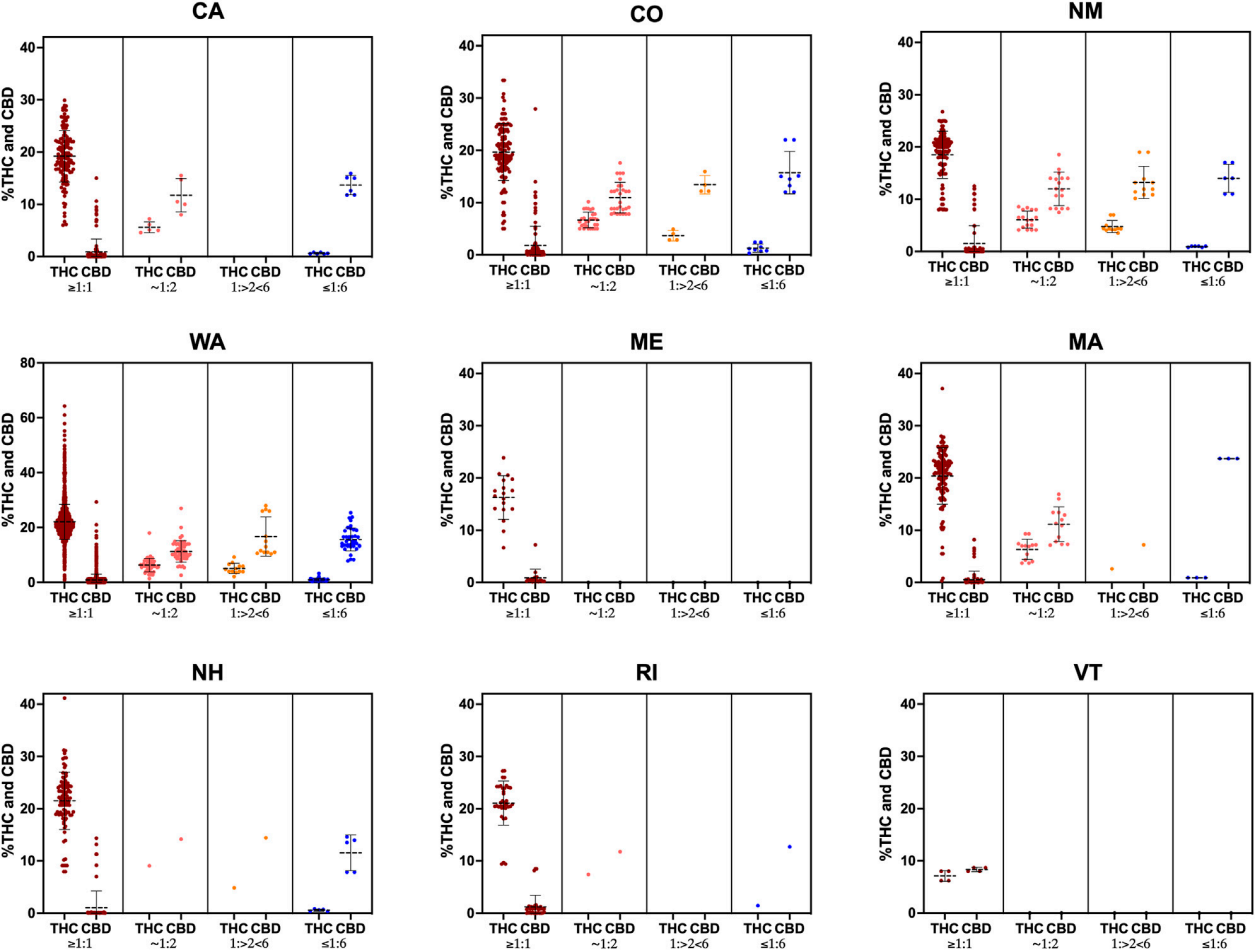
THC and CBD percent distribution by ratio category for all products with >0% CBD in each state. Data shown as mean ± SD.

Intriguingly, the majority of products within the THC:CBD ratios ≥1:1 category have >15% THC, a concentration that is highly intoxicating, in all states, with the exception of VT where products contain <10% THC (Figure 3). All other THC:CBD ratio categories are comprised of products with <10% THC in all studied states (Figure 3).

**FIGURE 4:**
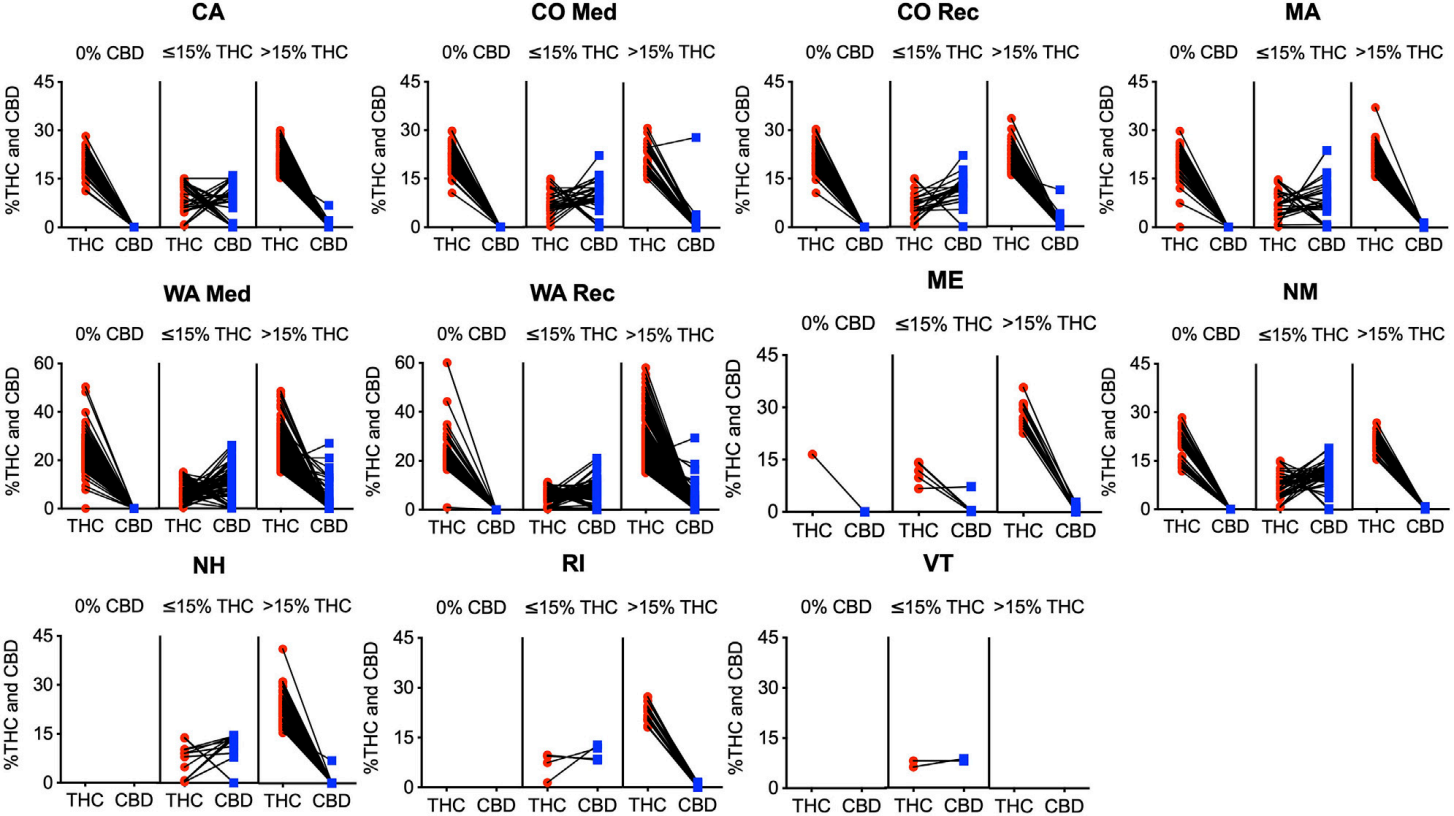
All individual products with CBD content information with corresponding THC and CBD percentage in each state. The first column for each graph contains all products with 0% CBD and their corresponding THC content; the second and third columns contain products with >0% CBD, with the second column containing products with ≤15% THC, and the third column containing products with >15% THC.

We observed that products with CBD information containing 0% CBD (839 products), have in average > 15% THC in all states with these products (NH, RI, and VT did not have this type of product); with NM, CA, CO (Medical and Recreational), and WA (Medical and Recreational) containing >20% and ME and MA containing 16.5 and 19.3% THC in average respectively (Figure 4). When products with CBD information containing <15% THC were examined (417 products deemed more suitable for medical purposes), we observed that the majority of products fall into the ∼ 1:2, 1:>2 < 6, and ≤1:6 ratio categories in all states except in ME where ≥1:1 products dominate; the THC average ranges from 6–9% and CBD averages from 6–11%, except in ME where THC and CBD averages were 11.7 and 1.4% respectively (Figure 4). Potent products with >15% THC and CBD information were very similar to products with 0% CBD, namely containing in average > 20% THC in all cases, except in ME (18% THC average), and VT (did not have products in this category), and <1% CBD levels in all states, except in CO Medical (1.5% CBD average; Figure 4).

## Discussion

Overall, this study’s results are alarming. They reveal current product offerings do not reflect scientific evidence regarding what concentrations of THC and CBD could be potentially therapeutic. Combined with holes in popular knowledge and misconceptions about THC and CBD, the current market can lead to problematic patient dosing as they try to maximize therapeutic benefits, such as analgesia, while subjecting themselves to THC’s acute intoxicating effects. For instance, across all states, the vast majority of both medical and recreational products with CBD (>0%) fall into the THC:CBD ratio category ≥1:1, with CBD likely enhancing THC subjective effects. The THC:CBD categories ≤1:6 and 1:>2 < 6, those with the lowest intoxication potential, provide the least amount of products. It is notable that products with lower THC, considered suitable for medical purposes, might in fact not have significant analgesic value (Dalton et al., 1976; van de Donk et al., 2019), since they have THC:CBD ratios of ≤1:6 or 1:>2 < 6, where CBD would likely reduce THC effects. More potent products that may be suitable for regular users or patients who have developed tolerance, those with 10–15% THC and ratios ≥1:1 and ∼ 1:2, are difficult to find in two major medical programs (CO and WA) when compared to >15% THC products. This leaves patients with mostly highly intoxicating options. Moreover, these findings are consistent across both medicinal and recreational programs, and in markets that offer both medical and recreational products (e.g., CA), or where all products are considered medical (e.g., NM). These findings also remain true regardless of whether they coexist in the same building (e.g., CO), or if they are in separated facilities (e.g., WA).

As shown, despite CBD having long been proven to pharmacologically alter cannabis’ overall effects, a large portion of products did not provide information on CBD content. This could potentially lead to unwanted side effects as patients do not have all the information on the drug they are taking. The results reveal that products with 0% CBD are very potent (Figure 4), with most products containing >15% THC, and virtually all containing close to or >10% THC. These products, especially those with >15% THC are counter indicated clinically and are therefore not recommended or safe to be marketed as medical cannabis (Romero-Sandoval et al., 2018; Boehnke et al., 2020; Cash et al., 2020). Similarly, products with CBD and >15% THC overwhelmingly behave similarly to those without any CBD. Virtually all of these high potency products contain <1% CBD, with mean values close to 0%. Consequently, products with high THC are likely to have little CBD. This theme can be helpful to note, especially for the significant number of products that do not offer information on CBD content. There are a few product exceptions in Washington medical and Colorado medical programs where there is more variation in CBD content, even in the high potency products. While there are certainly not enough of these outlying products to change the overall market makeup, this variation seems to indicate that medical programs recognize a demand for products different than those in the recreational market. Still, based on the literature, these products with high potency THC and high CBD concentrations likely produce significant unwanted psychotropic effects and can be harmful to patients seeking chronic pain relief (Wilsey et al., 2013; Andreae et al., 2015; Wallace et al., 2015; Boehnke et al., 2020; Pennypacker and Romero-Sandoval, 2020).

Beyond recent research demonstrating the effects of cannabis constituents, the momentum of current policy trends elicits a pressing need to understand the clinical therapeutic value of the cannabis available in the emerging market. As of February 2022, 37 states have legalized the medical usage of cannabis, and 18 states and Washington, D.C. have fully legalized cannabis (for both medical and recreational usage). Meanwhile, the rise of the opioid epidemic in U.S. has placed pain management under scrutiny and jumpstarted the search for treatment options with less adverse effects. Cannabis is advantageously place to be, and is often cited as an one of these alternatives (Caldera, 2020). In fact, presence of medical cannabis programs may be associated with a reduced opioid usage (Lucas, 2017). In the midst of the U.S. cannabis markets’ rapid evolution and the changing attitudes towards traditional pain management, fully understanding what cannabis products are offered from a pharmacologic perspective could both better inform patients and providers, and potentially shape usage and the future of the U.S. cannabis market.

We understand that our data show advertised products rather than consumer acquired products. However, our data matches the natural supply and demand dynamic of any commodity, for which cannabis is not an exception. Thus, the frequency of products identified in our study in terms of THC and CBD concentrations encompasses the frequency of product sales describe by others (Smart et al., 2017; Davenport, 2021). Furthermore, our data (frequency of potent herbal products) align with data on cannabis exposure from the National Poison Data System which shows that exposures more often involves plant material than other processed forms of cannabis products (e.g., edibles, concentrates, etc.) and this happens more often in states where adult cannabis use (recreational) is legal (Dilley et al., 2021). Similarly, it is important to note that the data used in this study was collected in 2018 (Cash et al., 2020). This is a limitation of the study as some of the data may have changed. However, the trends on market behavior this paper highlights are still relevant. If there are any pertinent changes, they are likely detrimental as the potency of cannabis has continually been increasing over the past several decades (ElSohly et al., 2016; Chandra et al., 2019; ElSohly et al., 2021). These themes are not limited to just the herbal market, but have been reflected in the edible cannabis market as well (Steigerwald et al., 2018). It is also relevant to highlight the expansion of the CBD product market. We do not know the extent to which CBD shops are influencing the presence of CBD in herbal cannabis products that have THC. According to trends found in illicit herbal cannabis products seized by the Drug Enforcement Administration, THC’s average potency ( ∼ 4% in 1995, 9.75% in 2009, and 13.88% in 2019) continues to rise and outpace CBD content ( ∼ 0.28% in 2001, 0.39% in 2009, and 0.56% 2019) (ElSohly et al., 2016; ElSohly et al., 2021). There was a substantial increase in the average THC:CBD ratio from 2009 to 2017 (24.81–103.48 respectively) which reversed in 2018 (54.39) and 2019 (24.58) (ElSohly et al., 2021). This reversal is potentially a result of the expanding legalization of marijuana and CBD product market, both of which should be reflected in this study’s data based on the timeframe. We therefore believe that this data is still highly relevant and reflective of the current market overall.

Furthermore, it is important to recognize that while this study’s results are concerning, they can also be seen as promising. In addition to the decreasing ratio recently noted, clinically meaningful options—those that can likely prove beneficial to patients—are offered in all states; they are just in the minority and need to be teased out. The hurdles ahead to salvage the medical cannabis market seem to be in two categories. First, changing public misconceptions about THC and CBD’s interplay and perceptions of what THC and CBD percentages clinically correlate too. Specifically, there is a need for education emphasizing that different concentrations of THC and CBD correlate to different pharmacologic effects, that adding high concentrations of CBD does not negate the psychotropic effects of THC, and that high potency cannabis (>15% THC) is in fact counter indicated for medical use. This will result in a more informed patient population. It can also help sway the demand away from high potency products and reduce incentives for the cannabis market to continually increase the potency of their offerings. Secondly, adequate policies regarding medical cannabis should also reflect the pharmacology and clinical correlates. This can be achieved through several means. By enforcing that products advertised for medical purposes actually have efficacy based on scientific literature, new policies can help expose the medically relevant products and segregate them from the recreational products. This could prove extremely meaningful, as it has been shown that patients regard the information provided by dispensaries as safe and reliable (Capler et al., 2017). Policies can also highlight the various clinically relevant ratios, fleshing out and offering substantial options in the therapeutically relevant categories. Lastly, policies can recommend dispensing medical products in a stepwise fashion, with the more potent products offered on a more stringent basis, such as after lower potency options proved ineffective for a patient. This can ensure an overall safer market for patients looking to achieve therapeutic benefits from cannabis without the risk of amplifying THC acute effects.

## Conclusion

In summary, medical cannabis programs’ mirror recreational cannabis programs’ herbal product offerings in terms of pharmacological profile, and do so regardless of facility type. An evaluation of these products’ ratios and concentrations revealed that the majority are highly potent (>15% THC) and contain THC:CBD ratios that will likely produce an additive effect on THC’s effects (≥1:1). And while analgesic effects likely parallel THC’s potency, so does intoxication and the frequency of adverse events (Wilsey et al., 2013; Andreae et al., 2015; Wallace et al., 2015; van de Donk et al., 2019). Therefore, many of the products marketed for medical purposes are counter indicated pharmacologically and potentially harmful (Romero-Sandoval et al., 2018; Boehnke et al., 2020). On the other hand, options that are likely the most suitable for therapeutic use are limited, even in medical programs. Ultimately, these results can be used to better inform patient populations and relevant policies and help steer the herbal medical cannabis market to be more reflective of clinical evidence.

## Data Availability

Publicly available datasets were analyzed in this study. This data can be found here: https://doi.org/10.1371/journal.pone.0230167.s018.
